# Draft Genome Sequence of *Thermus scotoductus* Strain K1, Isolated from a Geothermal Spring in Karvachar, Nagorno Karabakh

**DOI:** 10.1128/genomeA.01346-15

**Published:** 2015-11-12

**Authors:** Ani Saghatelyan, Lianna Poghosyan, Hovik Panosyan, Nils-Kåre Birkeland

**Affiliations:** a Department of Microbiology, Plants and Microbes Biotechnology, Yerevan State University, Yerevan, Armenia; b Department of Biology and Centre for Geobiology, University of Bergen, Bergen, Norway

## Abstract

The 2,379,636-bp draft genome sequence of *Thermus scotoductus* strain K1, isolated from geothermal spring outlet located in the Karvachar region in Nagorno Karabakh is presented. Strain K1 shares about 80% genome sequence similarity with *T. scotoductus* strain SA-01, recovered from a deep gold mine in South Africa.

## GENOME ANNOUNCEMENT

Bacteria belonging to the extremely thermophilic genus, *Thermus,* have been isolated from geothermal environments and compost with temperatures ranging from 55°C to 75°C (1). These microorganisms are of biotechnological interest due to their production of thermostable enzymes (2) and carotenoid antioxidants (3). They also serve as biological models for elucidating the mechanisms for biological adaptation to high temperatures (4). Although major advances have been made in the last decade, our knowledge of the physiology, geographic distribution, enzymology, and genetics of this group of organisms is still limited. Fifteen species belonging to this genus have been validly described, with *Thermus aquaticus* as the type species (5). Two strains belonging to the species *Thermus scotoductus* have been described; type strain SE-1, isolated from hot tap water in Iceland (6), and strain SA-01, isolated from fissure water with a temperature of about 60°C collected from a South African gold mine 3.2 km below surface (7). Only *T. scotoductus* strain SA-01 has been fully sequenced (8).

We have recently isolated a novel strain of *T. scotoductus* (designated K1) from sludge samples of a neutral 70°C geothermal spring outlet located in the Karvachar region of Nagorno Karabakh (40°17′41″ N, 46°27′50″ E). The strain was isolated on nutrient agar medium containing 0.1% tryptone and 0.1% yeast extract and subsequently cultivated in liquid medium containing 0.1% tryptone and 0.5% yeast extract. It grows optimally around 65°C and pH 8, is catalase and oxidase positive, and can grow anaerobically using nitrate respiration. Strain K1 shares >99% 16S rRNA sequence identity with strain SA-01. The draft genome of strain K1 was sequenced and assembled with PacBio RS technology and Celera Assembler, respectively, at GATC Biotech, Germany (http://www.gatc-biotech.com). A total of 110,643 reads, accounting for 303,436 kb of sequenced bases were obtained and assembled into 55 contigs comprising a total of 2,379,636 bp, which is slightly larger than the 2,346,803-bp genome of its closest relative, strain SA-01. The G+C content is 65.2%. Average nucleotide identity (ANI) analysis using the online ANI calculator (http://enve-omics.ce.gatech.edu/ani/index) and DNA:DNA hybridization analysis using the Genome-to-Genome Distance Calculator (http://ggdc.dsmz.de/distcalc2.php) revealed a two-way ANI value of 97.55% and 80.70% overall genome homology, respectively, between the K1and SA-01 strains, supporting their affiliation to the same species.

Gene prediction carried out with the NCBI Prokaryotic Genome Annotation Pipeline (9), as well as the RAST server (http://rast.nmpdr.org/rast.cgi), identified a total of 2,529 genes, including 2,104 coding DNA sequences, 3 sets of rRNA genes, 48 tRNA genes, and 2 ncRNA genes. The rRNA genes are unlinked, and located in separate 16S and 23/5S rRNA genes/operons, as is generally the case in *Thermus* spp. (10, 11). Two CRISPR arrays were identified. Genome-based knowledge of thermophilic microbes is of great importance and interest for assessing the diversity of enzymes of biotechnological importance and revealing their adaptation mechanisms to extreme conditions.

### Nucleotide sequence accession numbers.

This whole-genome shotgun project has been deposited at DDBJ/EMBL/GenBank under the accession number LJJR00000000. The version described in this paper is the first version, LJJR01000000.
